# *Fusarium equiseti* Pathogen of Potato in Kenya and Its Potential In Vitro Biocontrol Using Fungal Endophytes

**DOI:** 10.3390/pathogens14121239

**Published:** 2025-12-04

**Authors:** Marie Cecile Muhorakeye, Fathiya M. Khamis, Everlyne Samita Namikoye, Komivi S. Akutse

**Affiliations:** 1 International Centre of Insect Physiology and Ecology (ICIPE), Nairobi P.O. Box 30772-00100, Kenya; muhorakeyec64@gmail.com (M.C.M.); fkhamis@icipe.org (F.M.K.); 2 Department of Agricultural Science and Technology, Kenyatta University, Nairobi P.O. Box 43844-00100, Kenya; namikoye.samita@ku.ac.ke; 3 Department of Agricultural Engineering, Rwanda Polytechnic, Musanze College, Musanze P.O. Box 226, Rwanda; 4 Department of Zoology and Entomology, University of Pretoria, Private Bag X20, Hatfield 0028, South Africa; 5 Unit of Environmental Sciences and Management, North-West University, Private Bag X6001, Potchefstroom 2520, South Africa

**Keywords:** antifungal property, antagonistic fungi, dual culture assay, *Fusarium equiseti*, pathogenic fungi

## Abstract

Accurate pathogen diagnosis is fundamental for effective crop disease management. In Kenya, a pathogen causing significant damage in potato farms was initially misidentified as *Phytophthora infestans*. This study aimed to correctly identify this pathogen and explore initial control measures using a polyphasic approach. The methodology integrated morphological observation, pathogenicity testing, and molecular analysis using ITS and EF1-α gene sequencing. The results confirmed the pathogen’s identity as *Fusarium equiseti*, with morphological features consistent with this species and molecular sequencing showing 99.6% identity to reference strains. This is the first official report of *Fusarium equiseti* as a potato pathogen in Kenya. Furthermore, in vitro assays evaluated the efficacy of native fungal endophytes for biocontrol. Four endophytes inhibited the pathogen’s mycelial growth by over 70%, with *Trichoderma atroviride* isolate ICIPE 710 exhibiting the highest inhibition rate of approximately 91%. This research shows that effective identification of pathogens is crucial for proper management of diseases, ensuring timely control, saving resources and reducing crop losses. This study identified the native endophyte *Trichoderma atroviride* strain ICIPE 710 as a promising biocontrol candidate. The critical next step is to validate its efficacy in field trials for future development as a sustainable, practical alternative to chemical fungicides.

## 1. Introduction

Potato (*Solanum tuberosum* L.) is a key crop to Kenyans’ livelihood and the country’s economy. It is the second most produced crop, trailing maize and contributes to up to 500 million USD every year [1]. In addition, potatoes are one of the nine priority value chains, employing about 2.5 million people [2]. Despite the role of potatoes in ensuring food security, the crop production and productivity are obstructed by pests and diseases, of which potato cyst nematodes, bacterial wilt, and late blight diseases are the leading threats [2,3]. Aside from the mentioned well-known diseases, a fungal pathogen, “*Fusarium equiseti*”, causing wilting disease in potato, was recently discovered in a commercial farm located in the Ndabibi region in Naivasha town, Nakuru County, in Kenya, in mid-June 2022. Disease symptoms progressed systematically from localised infection to systemic plant collapse. Initially, the pathogen caused chlorosis and wilting of the lower leaves. As the infection advanced upwards, the upper leaves developed chlorosis. This was accompanied by the appearance of water-soaked lesions on the leaf margins and white sporulation on the abaxial (lower) leaf surfaces. Ultimately, these symptoms coalesced, leading to a generalised yellowing and wilting of the entire plant prior to death. Apparently, these symptoms were so similar to Late blight (barafu) disease of potato; however, the late blight invades plants starting from younger leaves. This makes people miss the real identity of the specific pathogen when referring to these symptoms, and it is therefore important to clearly identify the causal pathogen for its effective and sustainable management.

The invasion of potato farms by *Fusarium equiseti* had been reported in Poland [4] and in China [5]. This pathogen not only affects potato or other Solanaceae crops, but also crops from other families such as Leguminoseae, Poaceae, Cucurbitaceae, Apiaceae, and others [6]. *Fusarium equiseti* symptoms vary depending on the crop family affected; however, wilting, seed and root rotting, brownish discolouration and water-soaked lesions have often been observed [6,7,8,9]. According to Wang et al. [10] and Zhang et al. [11], this pathogen can form disease complexes with other pathogens, including viruses, and contributes to reduced yield quantity and quality. *Fusarium equiseti* significantly degrades produce quality by producing mycotoxins such as trichothecenes and Zearalenone, which were reported to have high toxic effects on human beings [6,12,13,14].

Endophytes are symbiotic microorganisms, such as bacteria and fungi, that live inside plant tissues without causing any disease symptoms [15,16,17]. In agriculture, they are known to enhance crop growth, nutrient uptake, and yield increase by improving the plant’s stress tolerance and disease resistance [18]. They achieve this through mechanisms such as modifying host cell walls, modulating phytohormones, secreting effector proteins, and producing antioxidants and other bioactive compounds that inhibit pathogens [17,19].

Frequently recognised antagonistic genera include *Pseudomonas*, *Enterobacter*, *Pantoea*, *Penicillium*, and *Trichoderma* [20]. Previous studies by Akutse, Agbessenou, and Paradza et al. [17,21,22,23] highlighted the potency of various *Hypocrea* and *Trichoderma* isolates against herbivorous pests, vectors and pathogens/diseases in tomato, nightshade, and beans. Furthermore, research by Muhorakeye et al. [17] previously demonstrated that these isolates can inhibit and suppress the mycelial growth of *Fusarium oxysporum f.sp. lycoperisici* [17]. Based on this known antagonistic efficacy, this study was initiated to explore their potential against the newly isolated *Fusarium equiseti*, a significant and novel threat to potato production in Kenya.

The emergence of *Fusarium equiseti* as a novel threat to potato production in Kenya necessitates the exploration of sustainable control strategies (our unpublished data). Building on the proven efficacy of native isolates against *Fusarium oxysporum* f.sp. *lycopersici* [17], this study aimed to evaluate their potential against this new pathogen. While biocontrol agents (BCAs) like *Trichoderma* and *Penicillium* species have shown promise against *F. equiseti* in other systems [14], their effectiveness is highly specific, varying not just by taxonomic group [9,24], but critically, by strain. This strain-specificity dictates the production of unique bioactive metabolites and colonisation capabilities, meaning successful biocontrol is a phenotype unique to individual strains. Therefore, this work specifically assessed the in vitro antagonistic potential of some selected native endophytic fungi, including *Trichoderma* spp. and *Hypocrea lixii*, for the sustainable management of *Fusarium equiseti*.

## 2. Materials and Methods

### 2.1. Sample Collection and Isolation of the Pathogen

**Figure 1:**
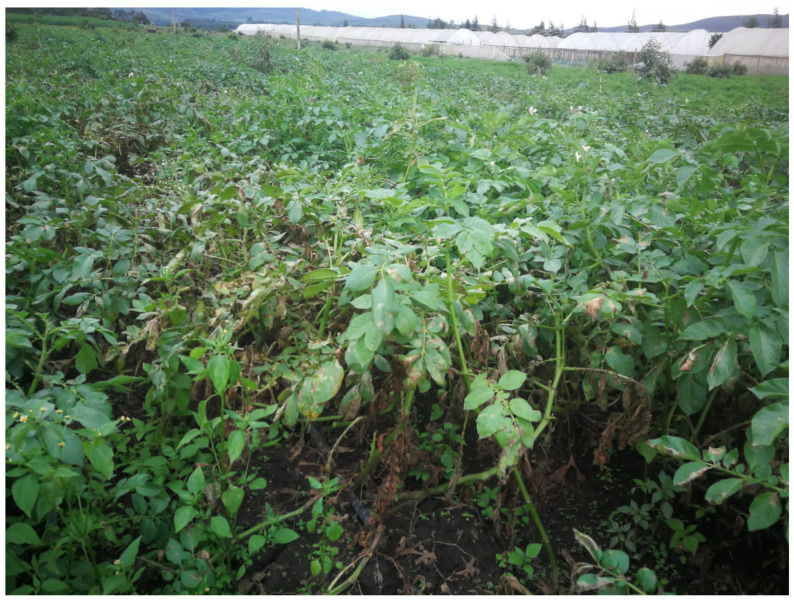
Observed symptoms of *Fusarium equiseti* on infected potato plants in the field at Naivasha, Kenya.

The experimental research and field studies on plants, including the collection of plant material, complied with relevant institutional, national, and international guidelines and legislation. The appropriate permissions and/or licenses for the collection of plant or seed specimens were obtained for the study as approved by the National Commission of Science, Technology and Innovations, Kenya (License No: NACOSTI/P/23/23608). The root, stem, and leaf samples were collected from infected potato plants (Figure 1) with dry-wilted, water-soaked lesions and white sporulation symptoms on their backside at two different farms in Ndabibi village of Naivasha, Nakuru County, Kenya (first field: latitude: −0.69222, S 0°41′31.992″, longitude 36.23775, E 36°14′15.912″ and second field: latitude −0.70143 S 0°42′5.142″, longitude: 36.21975, E 36°13′11.106″). The samples were placed in lunch boxes inside the cooler box and transported to the Arthropod Pathology Unit at the International Centre of Insect Physiology and Ecology (ICIPE), Nairobi, Kenya. The samples were washed with tap water and surface-sterilised by submersion in 70% ethanol for 1 min and 1.5% sodium hypochlorite for 2 min. Afterwards, they were washed three times in distilled water to remove excess sodium hypochlorite and blot-dried on a paper towel under a laminar flow hood. The samples were then cut into 1 × 1 cm pieces using sterile forceps and a scalpel. Three pieces were placed in a 90 mm Petri dish containing either Potato Dextrose Agar (PDA) (OXOID CM0139, Oxoid Ltd., Basingstoke, UK) (39 g/L) or V8 agar (200 mL of V8 juice, 3 g of CaCO_3_, 15 g of agar, and 800 mL of distilled water) media enriched with 40 μg/mL streptomycin sulfate antibiotic and incubated for 7 days at 25 ± 2 °C. The mycelial plug was then transferred into other Petri dishes with PDA or V8 agar media and incubated as above to obtain a pure culture through a series of subcultures.

### 2.2. Morphological Identification

The putative *Fusarium equiseti* was morphologically identified based on the colony growth and pigmentation it produced on both PDA and V8 agar media using the naked eye. Additionally, the isolate was identified based on the morphological features, such as type, shape, and size of spores produced using a microscope at 40× magnification in reference to the *Fusarium* spp. morphological key published by Leslie & Summerel [25].

### 2.3. Molecular Identification

Using a sterile scalpel, 1 g of fungal mycelia from a 2-week-old pure culture of the pathogen was harvested and placed into 2 mL sterile microcentrifuge tubes containing three glass beads (BioSpec Products Inc., Bartlesville, OK, USA). This was followed by DNA extraction using the Isolate II Plant DNA Extraction Kit (Meridian Bioscience, London, UK) in accordance with the manufacturer’s protocol. The quality and quantity of extracted DNA were evaluated using a NanoDrop™ 2000/2000c spectrophotometer Thermo Fischer Scientific, Wilmington, NC, USA). The samples were kept at −20 °C for further use. The molecular identification of *Fusarium* species was performed using both Intergenic Transcribed Spacer (*ITS*) region primers ITS 4 and ITS 5 [26] and elongation factor 1-α (*EF1α*) gene regions [27,28,29]. The PCR amplifications were performed in final total reaction volumes of 20 µL containing 5X *My Taq* Reaction Buffer (5 mM dNTPs, 15 mM MgCl_2_, stabilisers and enhancers), 0.5 pmol µL^−1^ of each primer, 0.5 mM MgCl_2_, 0.0625 U µL^−1^ *My Taq* DNA polymerase (Meridian Bioscience), and 15 ng µL^−1^ of DNA template. The reactions were set up in an Eppendorf Mastercycler^®^ Nexus Gradient Thermal Cycler (Eppendorf, Hamburg, Germany) with the following cycling conditions: initial denaturation for 2 min at 95 °C, followed by 40 cycles of 30 s at 95 °C, 45 s of annealing (59 °C for *ITS* and 58 °C for *EF1α*), and 1 min at 72 °C, then a final elongation step of 10 min at 72 °C. The PCR products were purified using the Isolate II PCR and Gel Kit (Meridian Bioscience) and sent for bi-directional Sanger sequencing at Macrogen Europe BV Laboratories (Meibergreef, Amsterdam, The Netherlands). The sequences obtained were assembled and edited using Geneious Version 8 [30,31] to generate consensus sequences. Afterwards, the consensus sequences were queried against publicly available reference sequences in the GenBank database hosted by the National Center for Biotechnology Information (NCBI).

Furthermore, phylogenetic inference of the sequences generated from this study and other *Fusarium* sp. sequences from the public database was performed using the Maximum Likelihood (ML) method under the Kimura 2-parameter model [30,31] where *Alternaria ventricosa* (PX307956.1) was included as the outgroup to root the tree. The tree is generated with the highest log-likelihood value of −2368.88, with branch support values expressed as the percentage of trees in which the associated taxa clustered together, are shown next to the corresponding nodes. The final phylogram is drawn to scale, with branch lengths representing the number of substitutions per site. A total of 804 nucleotide positions were included in the final dataset. All phylogenetic analyses were performed in MEGA X [32,33].

### 2.4. Pathogenicity Test

To confirm whether the isolated *Fusarium equiseti* was pathogenic, the pathogenicity test was conducted as described by Kearse et al. [34]. Certified potato seeds (tubers) of the famed Kenyan potato variety “Shangi” were obtained from the KALRO seeds unit, Kiambu county, Limuru-Tigoni area, Kenya. The tubers of uniform size (50–55 g) and bud number (4–5) were selected. Each tuber was planted in one of 16 pots (16.5 cm base diameter, 18 cm tall, and 22.5 cm top diameter), with eight replicates per treatment and arranged in a completely randomised design. The pots were filled with a 1:2:2 sterile mixture of topsoil, well-decomposed goat manure, and sand, respectively. This potting mixture had been autoclaved at 121 °C for 2 h and cooled for 72 h before use. In each pot, a potato tuber with at least 4 sprouts was planted in its centre and watered regularly every three days in the morning and kept inside the greenhouse at 12 h/12 h day/night photoperiod with 65% relative humidity. The inoculum was prepared as described by [35]. Once the seedling reached 8 weeks, eight pots were soil drenched with 10 mL of *Fusarium equiseti* inoculum at a concentration of 1 × 10^8^ conidia mL^−1^, while the remaining pots were soil drenched with 10 mL of distilled sterile Triton water and served as a control. The plants were monitored continuously until the symptoms of *Fusarium equiseti* disease appeared on infected potato, and roots, stem, and leaves samples were collected for re-isolation of the pathogen to prove Koch’s postulates [36].

### 2.5. Antagonistic Effect of Hypocrea lixii and Trichoderma spp. Against Fusarium equiseti

The in vitro antagonistic effects of the endophytic fungal isolates *Hypocrea lixii* F3ST1 and three *Trichoderma* spp. (*Trichoderma asperellum* M2RT4, *Trichoderma harzianum* KF2R41, and *Trichoderma atroviride* ICIPE 710) previously confirmed as endophytes in tomato and bean plants [17,23,37] were evaluated against the pathogen *Fusarium equiseti* using a dual culture assay following the modified methodology outlined by Morsy et al. [14]. All the tested fungal isolates were obtained from the *icipe* germplasm in Nairobi, Kenya. These isolates were subcultured on Potato Dextrose Agar (PDA) medium and incubated at 25 ± 2 °C in complete darkness for further use.

Prior to the bioassays, a spore viability test was performed to confirm that the selected fungal isolates are viable. Inoculum of all fungal isolates was prepared by harvesting conidia from the surface of two- to three-week-old sporulating cultures using a sterile spatula. The harvested conidia were mixed in 10 mL of sterile distilled water containing 0.05% (*w*/*v*) Triton X-100 (EMD Millipore Corporation, Burlington, MA, USA) in a universal bottle and vortexed for 5 min at about 700 rpm to produce homogenous conidial suspensions. Conidial counts were performed with an improved Neubauer Hemocytometer [21,22]. The inocula were adjusted to a concentration of 1 × 10^8^ conidia mL ^−1^. In addition, the conidia viability test was performed by spreading 0.1 mL of 3 × 10^6^ conidia mL ^−1^ onto 9 cm Petri dishes containing PDA. Plates were sealed with parafilm and incubated at 25 ± 2 °C in complete darkness for 15–18 h, depending on the fungal isolate, as some germinate faster than others [17,22]. The isolates were set in quadruplets and arranged in a completely randomised design. After 15–18 h, the plates were unsealed, fixed with a drop of fixative lactophenol cotton blue, and randomly covered with four sterile microscope coverslips (2 × 2 cm) [17,22]. Percentage conidial germination was determined from 100 randomly selected conidia on the surface covered by each coverslip under a light microscope (40×) [21,22]. A conidium was considered to have germinated when the length of the germ tube was at least twice its diameter [21,22]. The bioassay was performed in 90 mm Petri dishes containing solidified PDA or V8 agar. The bottom of each dish was marked on the exterior with a straight line dividing it into two equal compartments. Two points were marked on this line; each positioned 1 cm from the opposite edges of the dish. At each point, a 5 mm disc of agar was removed using a sterilised cork borer and an inoculating needle, creating two wells. Subsequently, 5 mm mycelial discs were aseptically excised from the margins of 7-day-old pure cultures of each antagonist and *Fusarium equiseti*. Using a sterile needle, a disc of the pathogen was placed face-up in one of the prepared wells. A disc of an antagonistic endophyte was placed in the opposite well. A control plate contained only the pathogen. Each treatment was replicated four times in a completely randomised design.

All plates were sealed and incubated at 25 ± 2 °C. The incubation continued until the mycelium of *Fusarium equiseti* had fully colonised the control plates. The percentage inhibition of radial growth (PIRG) was calculated to determine antagonistic activity, using the formula adopted from Myrchiang et al. [38]:

$$PIRG=\left[\left(R1-R2\right)/R1\right]\times 100,$$

where

- R1 is the radial growth of the pathogen from the inoculation points towards the opposite side of the plate in the control.
- R2 is the radial growth of the pathogen towards the antagonist in the dual culture plate.

### 2.6. Data Analysis

Before data analysis, the Shapiro–Wilk test was used to ensure that the data were normally distributed [39]. The data on inhibition of radial growth of *Fusarium equiseti* were analysed using a generalised linear model (GLM) package of R statistical software version 4.1.3 [40], assuming a binomial distribution with the log link function. The significance of the effects of endophytes on *Fusarium equiseti* mycelial growth inhibition was statistically assessed at a *p* < 0.05 significance level. For any substantial variations among treatments, the Student Newman Keuls (SNK) test was used to separate the means.

## 3. Results

### 3.1. Macroscopic Identification of Fusarium equiseti

**Figure 2:**
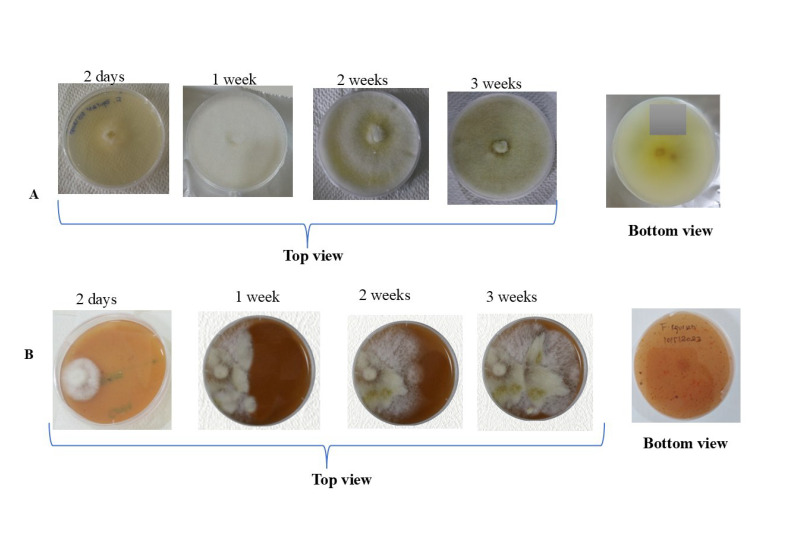
Growth of *Fusarium equiseti* on: (**A**) PDA medium; (**B**) V8 agar medium.

The putative *Fusarium equiseti* successfully grew on both V8 agar and PDA media. From the top view of Petri dishes with PDA medium, *Fusarium equiseti* produced profuse milky white mycelia, of which a yellowish to golden ring appeared in the centre of the plate after a week. As the culture aged, the yellow colour turned pale to dark brown, while a white-greenish yellow pigmentation was observed at the bottom side of the Petri dish (Figure 2A). On the other hand, the observed growth characteristics of *Fusarium equiseti* on V8 agar medium were the same as those reported on PDA; however, at the bottom side of the plates with V8 agar medium, no pigmentation was observed (Figure 2B).

### 3.2. Microscopic Identification of Fusarium equiseti

**Figure 3:**
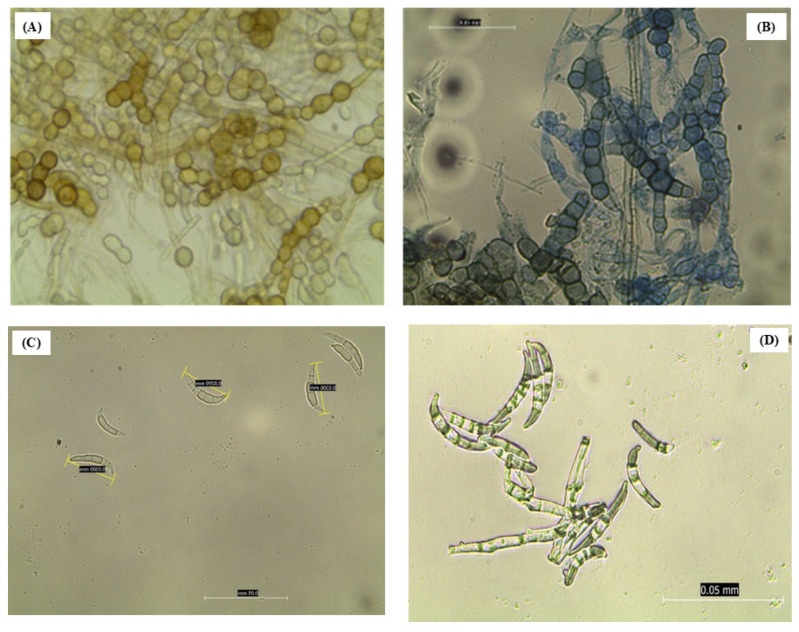
Morphological observations or features of *Fusarium equiseti* under the microscope at ×40 magnification. (**A**,**B**) Chlamydospores, (**C**,**D**) Macroconidia.

Under the microscope, a huge mass of hyphae containing chains of chlamydospores stained in yellowish-brown pigment was observed after 2 weeks post-incubation (Figure 3A,B). By the third week of incubation, orange sporodochia had developed macroconidia originating from monophialides on branched conidiophores possessing elongated, tapered apical cells. These macroconidia displayed a slender, curved morphology with gradual tapering at both ends, averaging 30 µm in length and typically exhibiting five (5) clearly visible septa (Figure 3C). In the fourth and fifth weeks, the macroconidia were completely visible and increased in number (Figure 3D). However, no microconidia were observed (Figure 3).

### 3.3. Molecular Identification of the Pathogen

**Figure 4:**
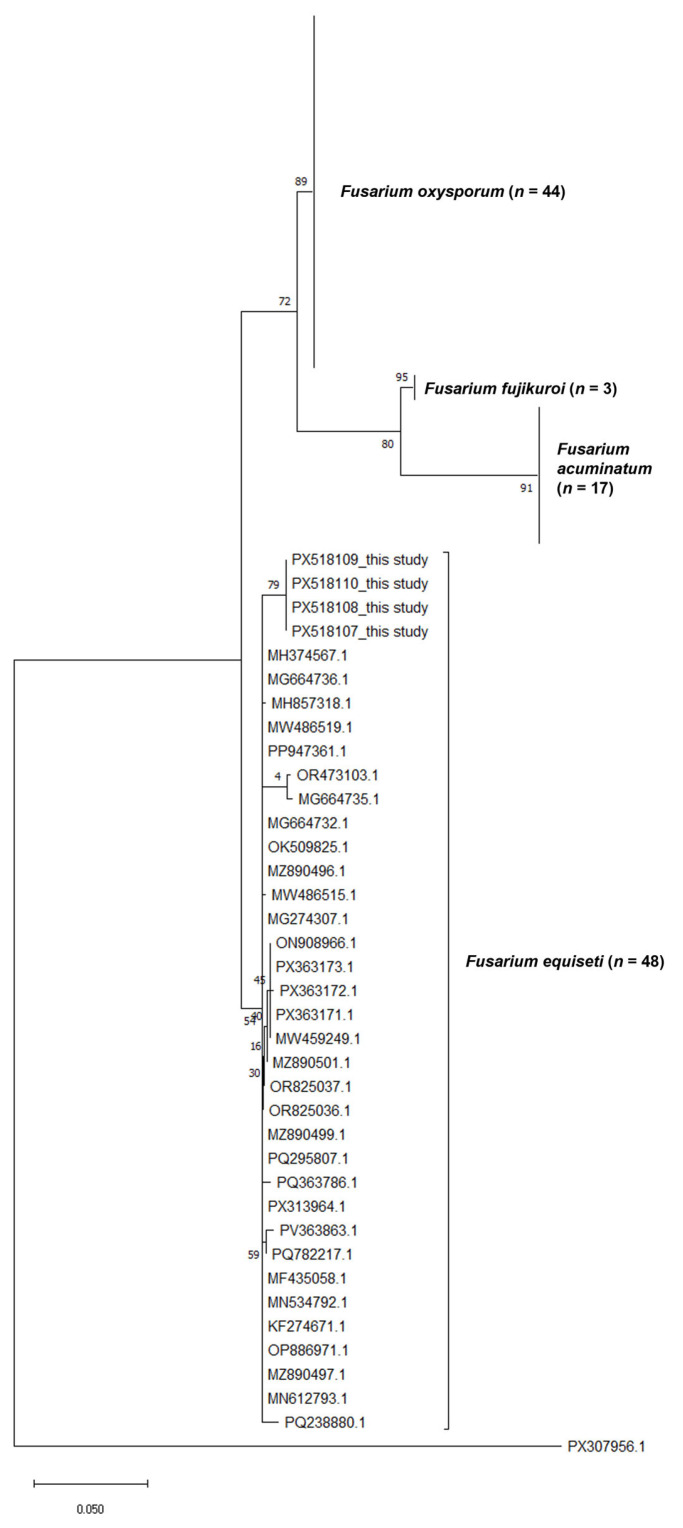
Maximum Likelihood phylogenetic tree based on the *ITS* gene region of *Fusarium equiseti* and other related *Fusarium* species.

Besides morphological identification of the pathogen as *Fusarium equiseti,* the samples were further confirmed by sequences of the *ITS* and *EF1α* gene regions obtained from the study. The resultant *ITS* and *EF1α* sequences had 99.6% and 94.84–99.61% identity similarity match, respectively, with publicly available *Fusarium equiseti* sequences (MG664739.1 and MK278902.1). Furthermore, the sequences of the identified phytopathogen were subsequently deposited to GenBank and assigned accessions PX518107–PX518110 and PX548786–PX548787, for *ITS* and *EF1α* gene regions, respectively. Subsequently, a phylogenetic tree (Figure 4) was constructed based on the *ITS* gene region of the sequences generated from this study and using 112 *Fusarium* spp., dataset from GenBank. The isolates generated in this study (PX518107–PX518110) clustered tightly within the *Fusarium equiseti* clade, together with multiple reference sequences representing the species. This cluster is strongly supported by high bootstrap values, indicating that the study isolates are genetically congruent with authenticated *Fusarium equiseti* sequences. Other *Fusarium* species formed distinct, well-resolved clades separate from *Fusarium equiseti*. *Fusarium oxysporum* (*n* = 44) grouped into a large, coherent lineage with strong branch support, reflecting its well-characterised diversity. Similarly, *Fusarium fujikuroi* (*n* = 3) and *Fusarium acuminatum* (*n* = 17) each formed independent clusters with high bootstrap support, demonstrating clear phylogenetic distinction from the *Fusarium. equiseti* clade (Figure 4).

### 3.4. Pathogenicity Test for Fusarium equiseti

**Figure 5:**
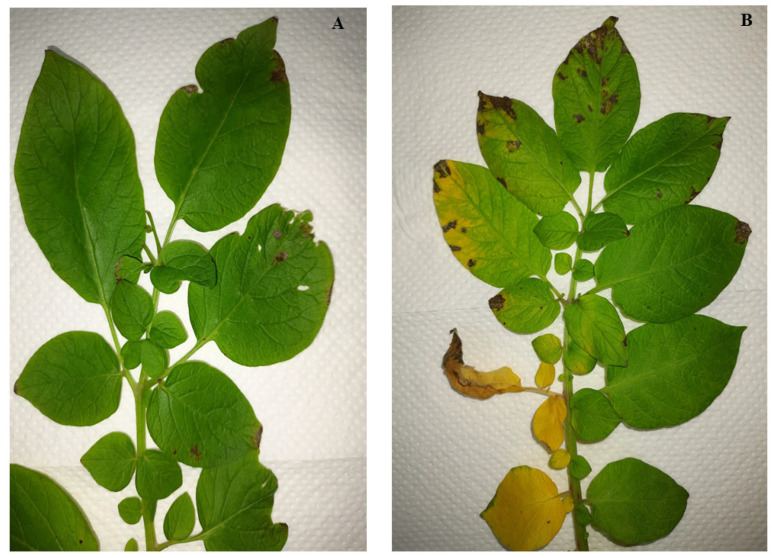
Symptoms of the isolated *Fusarium equiseti* on potato plant leaf after re-infection: (**A**) Control; (**B**) Inoculated.

A significant difference (*p* < 0.001) was observed between *Fusarium equiseti*-infected and control potato plants. The results of the re-infection test showed that 87.5% (seven out of eight) potato plants inoculated with *Fusarium equiseti* began to display disease symptoms by the 2nd week after inoculation. The symptoms progressed through the fourth and fifth week, by which time most parts of the inoculated plants were affected. The primary symptoms observed were wilting and chlorosis of the lower leaves, which subsequently progressed to the upper leaves. These symptoms intensified until one side of the plant or the entire plant became yellowish (Figure 5). Furthermore, the re-isolated pathogen exhibited morphological traits similar to the original colony, and the macroconidia and chlamydospores observed matched those previously obtained from the initial isolation. Thus, the findings confirmed that the isolated *Fusarium equiseti* was virulent in the potato variety “Shangi”.

### 3.5. Antifungal Activity of Some Selected Endophytic Fungal Isolates Against Fusarium equiseti

**Figure 6:**
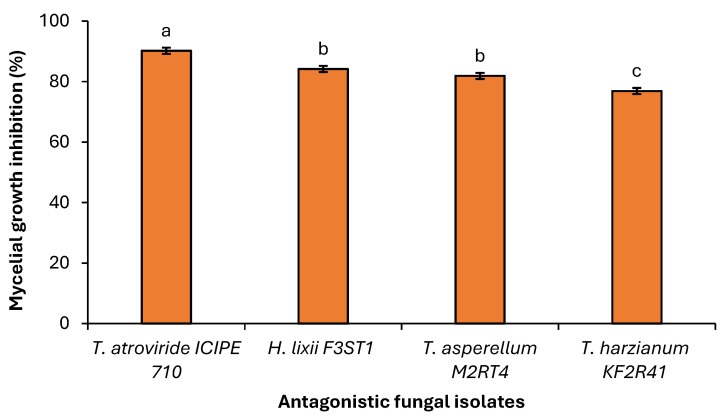
Average percentage of mycelial growth inhibition of three *Trichoderma* spp. and *Hypocrea lixii* F3ST1 against *Fusarium equiseti* on V8 agar medium. Different small letters above the error bars reflect significant differences across the treatments.

**Figure 7:**
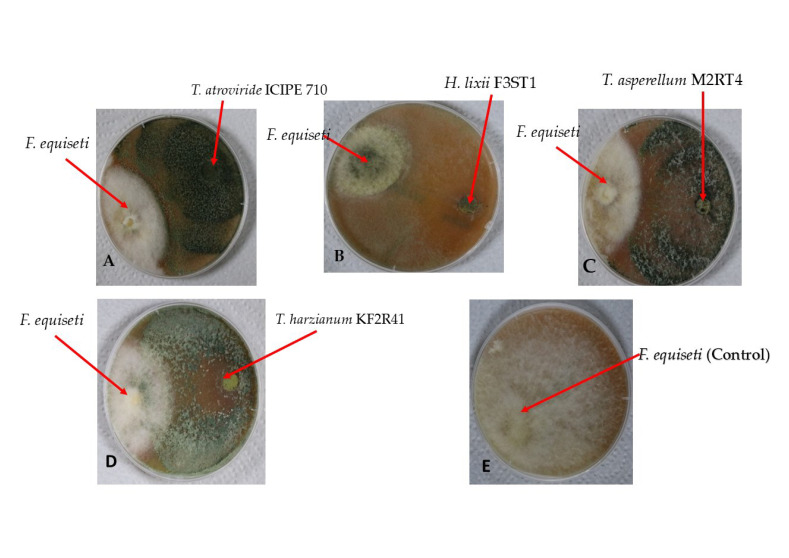
Antifungal activity of four endophytic fungal isolates against *Fusarium equiseti* mycelium growth on V8 agar media, on the 9th day of their dual culture assay: (**A**) *Trichoderma atroviride* ICIPE 710; (**B**) *Hypocrea lixii* F3ST1; (**C**) *Trichoderma asperellum* M2RT4; (**D**) *Trichoderma harzianum* KF2R41; and (**E**) Control.

A significant difference (χ^2^ = 40.253, df = 3, *p* < 0.001) was observed among the selected endophytic fungal isolates against the *Fusarium equiseti* phytofungus with regard to the radial growth or inhibition rates on the 9th day of their dual culture on V8 agar medium (Figure 6). Among the evaluated endophytes, *Trichoderma atroviride* ICIPE 710 exhibited the highest inhibition (90.23%) of *Fusarium equiseti* mycelium growth, followed by *Hypocrea lixii* F3ST1 (84.23%), *Trichoderma. asperellum* M2RT4 (81.92%), while *Trichoderma harzianum* KF2R41 recorded the lowest *Fusarium equiseti* mycelial growth inhibition (76.92%) (Figure 7).

**Figure 8:**
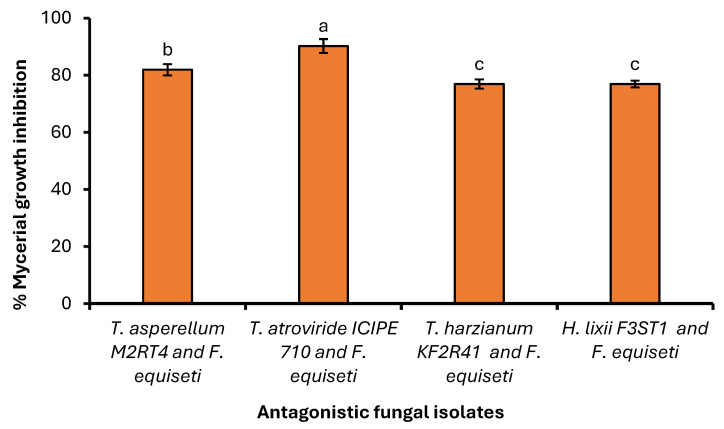
Average percentage (Mean ± SE) of mycelial growth inhibition of three *Trichoderma* spp. and *Hypocrea lixii* F3ST1 against *Fusarium equiseti* on PDA medium. Different small letters above the error bars reflect significant differences across the treatments.

**Figure 9:**
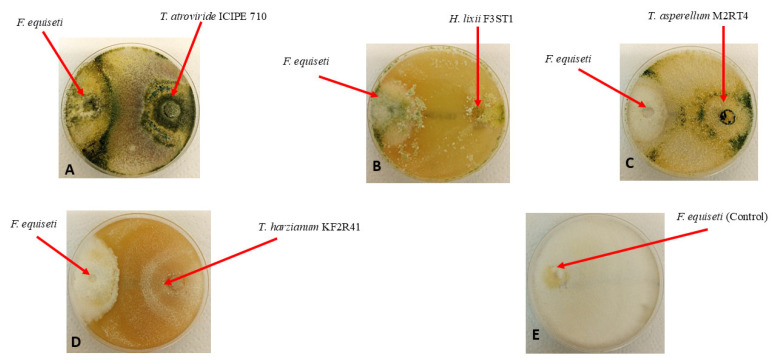
Antifungal activity of four endophytic fungal isolates against *Fusarium equiseti* mycelium growth on PDA media, on the 13th day of their dual culture assay: (**A**) *Trichoderma atroviride* ICIPE 710; (**B**) *Hypocrea lixii* F3ST1; (**C**) *Trichoderma asperellum* M2RT4; (**D**) *Trichoderma harzianum* KF2R41; and (**E**) Control.

Similarly, the antagonist assay of the above-selected endophytes against *Fusarium equiseti* was conducted on PDA medium with similar effects on the phytopathogen. A significant difference (χ^2^ = 57.524, df = 3, *p* < 0.001) was observed among the selected endophytes against *Fusarium equiseti* 13th day post-inoculation (dual culture) when control plates well fully covered with *Fusarium equiseti* mycelia (Figure 8). Endophytes *Trichoderma atroviride* ICIPE 710, *Hypocrea lixii* F3ST1, *Trichoderma asperellum* M2RT4, and *Trichoderma harzianum* KF2R41 achieved 90.98%, 84.30%, 82.70%, and 77.59% inhibition rates against *Fusarium equiseti*, respectively (Figure 9).

## 4. Discussion

This study presents the first documented evidence of *Fusarium equiseti* as a fungal pathogen causing wilting and blight-like symptoms in potato crops in Naivasha, Kenya. This finding clarifies a significant diagnostic discrepancy in the region. While field observations initially suggested *Phytophthora infestans*, evidenced by symptoms like brown water-soaked lesions and white sporulation on the lower leaves, comprehensive laboratory analysis consistently demonstrated *Fusarium equiseti* as the sole causal agent. This clarifies the prevailing misconception among local farmers, who commonly attribute the symptoms to late blight (barafu). The occurrence of blight-like symptoms may be influenced by the local climate, whereas the predominant wilting observed in unshaded areas is a more typical presence of *Fusarium* infection. These findings concurred with previous studies that have identified *Fusarium equiseti* as a pathogen causing similar blight and wilt symptoms in other hosts, including those in alfalfa and potato [5,6].

In this study, morphological and molecular tools, along with pathogenicity tests, confirmed the identity of the pathogen as *Fusarium equiseti* and its role in causing the disease. Profuse milky white mycelia with a yellowish ring that turns to pale to brown with time, together with slender, curved and tapered at both ends macroconidia with five septa, were morphological features that were observed in the isolated fungus. These features were consistent with previous findings reported by [7,25,29,41]. Moreover, the phylogenetic reconstruction based on the ITS gene region provides strong support for the morphological and molecular identification of the pathogen in the *Fusarium equiseti* with minimal divergence from authenticated *F. equiseti* accessions, forming a tight and well-supported cluster within the *Fusarium equiseti* lineage. This high degree of genetic concordance reinforces the accuracy of the species-level identification [32,42]. The clear separation of *Fusarium equiseti* from other closely related *Fusarium* species, including *F. oxysporum*, *F. fujikuroi*, and *F. acuminatum*, demonstrates the robustness of the *ITS* gene region for resolving species boundaries within the genus, particularly when paired with curated reference datasets [43]. The distinct clustering patterns observed in the phylogram reflect well-established evolutionary relationships among *Fusarium* species complexes and highlight the taxonomic coherence and stability of the *Fusarium equiseti* clade [42]. Additionally, the inclusion of *Alternaria ventricosa* as the outgroup was instrumental in reliably rooting the phylogenetic tree. By anchoring the analysis outside the genus *Fusarium*, the outgroup enabled accurate interpretation of lineage divergence and species-level clustering, ensuring the correct placement of the *Fusarium equiseti* isolates within their evolutionary context [26]. Consequently, this conclusively identified the phytopathogen, which was causing the described symptoms that occurred at the sampled potato fields.

Having identified *Fusarium equiseti* as a novel and significant pathogen, the development of sustainable management strategies becomes imperative. Current control methods for *Fusarium* spp. in potatoes, such as chemical fungicides, nanoparticles, and RNA interference (RNAi), are often hampered by issues of fungicide resistance, questionable environmental safety, and high costs that limit accessibility for smallholder farmers [20,44,45]. In light of these challenges, the use of beneficial antagonistic microorganisms emerges as one of the most promising, safe, and sustainable solutions [14,20].

Among the most frequently recognised antagonists are genera such as *Pseudomonas*, *Enterobacter*, *Pantoea*, *Penicillium*, and *Trichoderma* [20], which can protect plants through endophytic colonisation, antibiosis, and mycoparasitism. This provides a clear rationale and evidence for the high antagonistic potential observed in our study. The results of this study confirm the potent biocontrol ability of the native fungal endophytes *Hypocrea lixii* F3ST1, *Trichoderma atroviride* ICIPE710, and *Trichoderma asperellum* M2RT4 and *Trichoderma harzianum* KF2R41 against *Fusarium equiseti*. All assessed isolates effectively inhibited the pathogen’s growth in vitro by more than 70% on both PDA and V8 media, a finding which is consistent with the established efficacy of these genera against *Fusarium* spp. [9,17,46,47,48]. This strong inhibition could be mediated by well-documented mechanisms such as competition and antibiosis [17,46]. However, it is important to validate these findings in the field efficacy trials through *in planta* assessment for potential formulations and deployment for sustainable management of *Fusarium equiseti* in potato cropping systems.

## 5. Conclusions

This study provides the first confirmed report of *Fusarium equiseti* as a causal agent of wilt disease in potato (*Solanum tuberosum* L.) in Kenya. Furthermore, this research identifies a potentially sustainable management strategy by demonstrating the marked in vitro antagonism of native endophytic fungi against this pathogen. Among the isolates evaluated, *Trichoderma atroviride* ICIPE 710 outperformed all the other fungal isolates by significantly inhibiting the mycelial growth of *Fusarium equiseti* on both PDA and V8 agar media. Consequently, this isolate could therefore represent a promising candidate for further investigation as an effective biocontrol agent against this phytopathogen. However, these findings are derived solely from the in vitro assays and require further efficacy studies. The efficacy of *Trichoderma atroviride* ICIPE 710 must therefore be validated through in vivo greenhouse and field trials before any practical application can be recommended. Future studies should also investigate the influence of environmental factors on this disease’s expression. For instance, the cold, humid conditions of the Naivasha region may cause *Fusarium equiseti* to manifest symptoms that could be easily confused with late blight (*Phytophthora infestans*), complicating field diagnosis. Additionally, a broader survey to determine the geographic distribution and prevalence of *Fusarium equiseti* across Kenya’s potato-growing areas is warranted to inform targeted phytosanitary measures and mitigate its further spread.

## Data Availability

The data generated during the current study are available from the corresponding author upon request.
